# Programmable structured surfaces can change the future of wireless communications

**DOI:** 10.1038/s41377-022-00878-6

**Published:** 2022-06-28

**Authors:** Lei Xu, Mohsen Rahmani

**Affiliations:** Advanced Optics and Photonics Laboratory, Department of Engineering, School of Science and Technology, Nottingham Trent University, Nottingham, NG11 8NS UK

**Keywords:** Microwave photonics, Optoelectronic devices and components

## Abstract

An innovative time-varying metasurface has been reported to realise dual-channel data transmissions for light-to-microwave signal conversion. Such a novel technique is a remarkable step forward to realise full-spectrum networks for catering for the growing demand for wireless communications. Moreover, this technique enriches the functionalities of tunable metasurfaces and stimulates new information-oriented applications.

Wireless communication is a fast-growing and vibrant segment within the communications industry. It plays an increasingly important role in our daily lives, owing to its robust ability to transmit data without using any connections such as wires, cables, or other physical media to guide signal propagation. Currently, the deployed networks, using radio frequency (RF) communications, are characterised by a shared medium, limited spectrum bandwidth, and a lack of ability to scale with an increasing number of end devices^1^. Therefore, today’s technology does not cater to the growing demand for wireless data. To overcome these limitations, there is a quest to employ multi-domain communication systems, so-called hybrid systems, that can exploit other domains, such as terahertz^2^, microwave^3^, and optical^4^ electromagnetic (EM) waves. Such hybrid systems sound the best approach to realise full-spectrum networks for future sixth generation (6G) wireless communications.

The optical spectrum is considered a promising communication resource among various communication domains due to its vast license-free spectrum range, high security, high-energy efficiency, and electromagnetic interference-free fashion^4^. Furthermore, it is perfectly suitable for many unique application scenarios, such as indoor communication, underwater communications, and data transfer in some EM-sensitive environments, including medical facilities, hospitals, and underground mines^4^. However, the current technological challenge to fully taking the optical domain on board is converting it to the other domains with minimal loss. Traditionally, such a function requires complicated relaying systems where the received optical signals are firstly amplified and converted to baseband before being down-converted to the other domains. Such an approach needs a large number of additional hardware and multiple process steps causing further delays, higher costs, and more energy resources.

Writing in this issue of Light: Science & Applications, Tie Jun Cui and co-workers at Southeast University (China), Purple Mountain Laboratories (China), and the National University of Singapore have developed an innovative transmitter for direct signal conversion from visible lights to microwaves. They have employed the concept of metasurfaces, i.e. two-dimensional (2D) structured surfaces with precisely engineered elements in subwavelength scales. Their proposed transmitter can directly convert the real-time signal from visible lights to microwaves and facilitate promising hybrid wireless communication^5^.

Metasurfaces are artificial arrays of subwavelength unit cells^6–8^ that can be used to manipulate EM radiation in unconventional ways to offer exotic applications, such as metalenses, beam shapers, holograms, nonlinear metasurfaces, and remote quantum control^9–13^. Programmable metasurfaces are an emerging sub-category of metasurfaces that allow EM manipulation and information processing dynamically^14–16^. Such metasurfaces, mostly electrically controlled, have opened up many attractive functions, such as Doppler cloaking^17^, frequency conversion^18^, and information processing^19^. However, the electrical control method limits the programmable metasurfaces to be only considered in a single domain and not applicable to wireless communications.

The authors have designed, fabricated, and demonstrated a metasurface-based transmitter that is low-cost, low-complexity, wireless and multi-domain. Their device consists of a time-varying microwave metasurface and a photoelectric detection circuit sensitive to light. It has been demonstrated that the reflection frequency of the metasurface platform can be controlled by the light intensity waveform in real time. In other words, the programable metasurface can convert a light intensity signal to two microwave binary frequency shift keying signals directly, achieving the dual-channel data transmissions in a light-to-microwave link (see Fig. 1). Such a wireless communication system can transmit two different videos simultaneously using a frequency division multiplexing (FDM) scheme.

**Fig. 1:**
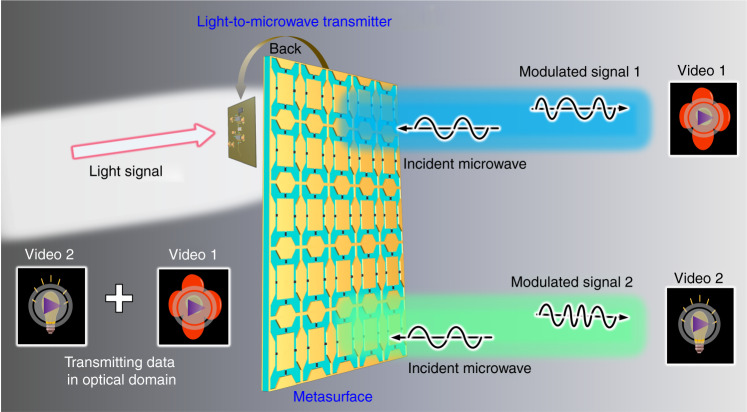
Schematic of direct light-to-microwave signal conversion using a hybrid optically programmed time-varying metasurface platform. A dual-channel light-to-microwave wireless link is built up via the metasurface-enabled signal converter. In such a system, two different videos can be transmitted and received simultaneously and independently.

The demonstrated light-to-microwave signal conversion process was completed fully on a sole platform, without additional RF devices and optical components. Such a platform offers low-cost and low-complexity hybrid communication systems and opens the promising potential for future multi-domain integrated 6G, a new era of wireless communications. In addition, this technique will enrich the functionalities of metasurfaces, and could also stimulate new information-oriented applications.
